# 

**Published:** 1868

**Authors:** 


### CONTENTS.

### NUMBER II.

### ORIGINAL COMMUNICATIONS.

PAGE.

ARTICLE I.—A Case of Pleuro-Pneumonia, Associated with Intermittent Fever. By Wm. O'Daniel, M. D., Marion, Ga. .... 65

ARTICLE II.—Uterine Diseases. By J. G. Westmoreland, M. D., Professor of Materia Medica and Therapeutics in Atlanta Medical College .... 68

ARTICLE III.—European Correspondence .... 76

ARTICLE IV.—European Correspondence .... 83

### SELECTIONS.

ARTICLE V.—The Sulphites in Typhoid or Enteric Fever. By R. S. Cross, Esq., M.R.C.S., L.S.A. .... 87
ARTICLE VI.—An Address on the Present State of Therapeutics.—Delivered at the Opening Meeting of the Clinical Society of London. By Sir Thomas Watson, Bart., M. D. .... 96
ARTICLE VII.—Wood's Operations for Radical Cure of Hernia. By Benjamin Howard, M. D., Lecturer on Operative and Minor Surgery in the Medical College of the University of New York .... 100
ARTICLE VIII.—Colica Pictonum—Alcohol its Exciting Cause—The Abuse of Lead. By Amos Sawyer, M. D., Hillsboro, Ill. .... 103

### EDITORIAL AND MISCELLANEOUS.

Bibliographical .... 106
College Dispensary .... 108
Prof. J. Boring .... 111
Atlanta Medical College. L. P. Yandell, Jr., M. D. .... 113
Richmond Medical Journal .... 114
Impurities in Glycerin. Syrup of Iodide of Iron .... 115
Carbolic Acid in Burns. Medicated Bougies for Gonorrhœa. Physiological and Therapeutical Action of Caffein .... 116
Influence Exerted by Anæsthetics on the Brain and Nervous System .... 117
Action of Putrid Material on the Animal Organism .... 119
Zymosis—A New Antiseptic Salt .... 120
Sore Nipples. Ulceration of the Tonsils. Chloroform in Intermittent Fever .... 121
Enlargement of the Lymphatic Glands .... 122
Punishment of Abortionists .... 124
A New Invalid Bed .... 125
Chronology of Anæsthesia. Subscription Receipts for the Journal .... 128

